# Patient-specific molecular alterations are associated with metastatic clear cell renal cell cancer progressing under tyrosine kinase inhibitor therapy

**DOI:** 10.18632/oncotarget.18200

**Published:** 2017-05-23

**Authors:** Steffen Dietz, Holger Sültmann, YueJun Du, Eva Reisinger, Anja Lisa Riediger, Anna-Lena Volckmar, Albrecht Stenzinger, Matthias Schlesner, Dirk Jäger, Markus Hohenfellner, Stefan Duensing, Carsten Grüllich, Sascha Pahernik

**Affiliations:** ^1^ Cancer Genome Research Group, German Cancer Consortium (DKTK), Heidelberg, Germany, German Cancer Research Center (DKFZ) and National Center for Tumor Diseases (NCT), Heidelberg, Germany; ^2^ Department of Urology, Heidelberg University Hospital, Heidelberg, Germany; ^3^ Department of Urology, Nanfang Hospital of Southern Medical University, Guangzhou, China; ^4^ Data Management Group, Division of Theoretical Bioinformatics, German Cancer Research Center (DKFZ), and Heidelberg Center for Personalized Oncology (DKFZ-HIPO), Heidelberg, Germany; ^5^ Department of Medical Oncology, National Center for Tumor Diseases (NCT), Heidelberg University Hospital, Heidelberg, Germany; ^6^ Institute of Pathology, University Hospital Heidelberg, and German Cancer Consortium (DKTK), Heidelberg, Germany; ^7^ Division of Theoretical Bioinformatics, German Cancer Research Center (DKFZ), Heidelberg, Germany; ^8^ Section of Molecular Urooncology, Department of Urology, Heidelberg University Hospital, Heidelberg, Germany; ^9^ Department of Urology, Nuremberg General Hospital, Paracelsus Medical University, Nuremberg, Germany

**Keywords:** clonal evolution, metastatic clear cell renal cell carcinoma, next generation sequencing, therapy resistance, tyrosine kinase inhibitors

## Abstract

The availability of tyrosine kinase inhibitors (TKI) during the past ten years has led to improved response and overall survival of patients suffering from metastatic clear cell renal cell carcinoma (ccRCC). However, most of these tumors will eventually progress due to resistance evolving under therapy. The objective of this pilot study was to determine whether molecular alterations in ccRCC tissues sampled over the course of the disease might be suggestive of potential therapies. We performed whole exome sequencing of nine samples from four patients in the MORE (Molecular Renal Cancer Evolution) trial. We analyzed the mutational patterns in the tissues at baseline and compared them to those detectable in biopsy samples after progression under TKI therapy. We found limited genetic concordance between primary and secondary tumor sites with private mutations in *FLT4, MTOR, ITGA5, SETD2, PBRM1*, and *BRCA1* on progression. One patient who showed an increased mutational load in the metastasis responded to nivolumab treatment. Our data provide evidence for clonal evolution and diverse pathways leading to acquired TKI resistance of ccRCC. Acquired resistance to TKI in metastatic ccRCC is due to intra-tumor heterogeneity and clonal evolution of resistant subclones. Mutations occurring under progression might be informative for alternative targeted therapies.

## INTRODUCTION

Kidney cancer represents one of the most frequent malignant neoplasms in the United States [1]. About 80% of kidney tumors belong to the clear cell renal cell carcinoma (ccRCC) histology. Thirty percent of ccRCC patients develop metastatic disease (mRCC), which is associated with poor prognosis and short overall survival (OS) [2]. However, since the approval of the first targeted drug (sorafenib) in 2005, the therapeutic landscape has changed considerably, and OS of mRCC increased from nine months in 2005 to 30 months in 2011 [3]. Today, approved targeted drugs for ccRCC include tyrosine-kinase inhibitors (TKI), mTOR pathway inhibitors and antibodies against the vascular endothelial growth factor (VEGF). In particular, TKIs such as sunitinib, which target multiple receptors at the same time, have been highly successful in the treatment of mRCC patients [4]. Currently, ten different drugs are approved for first- and second-line therapy [5–10]. The efficacy of these agents is highest in the first-line setting with the choice of agent class being important for OS [11]. However, the optimal sequence of targeted drugs is unknown due to a lack of biomarkers for patient stratification [12, 13]. On the molecular level, ccRCC is a heterogeneous tumor displaying a broad spectrum of genetic alterations [14, 15]. Multiregion sequencing revealed intra-tumor heterogeneity of mRCC with wide subclonal diversity [16–18]. Here, we report the results of a pilot study (MORE, Molecular Renal Cancer Evolution) using whole-exome sequencing (WES) of primary and biopsy tissue samples from treatment-naïve patients at baseline and after TKI treatment respectively. Our aim was to assess whether genetic alterations that can be exploited therapeutically develop in metastatic sites during disease progression under TKI regimens.

## RESULTS

### Patients

**Figure 1:**
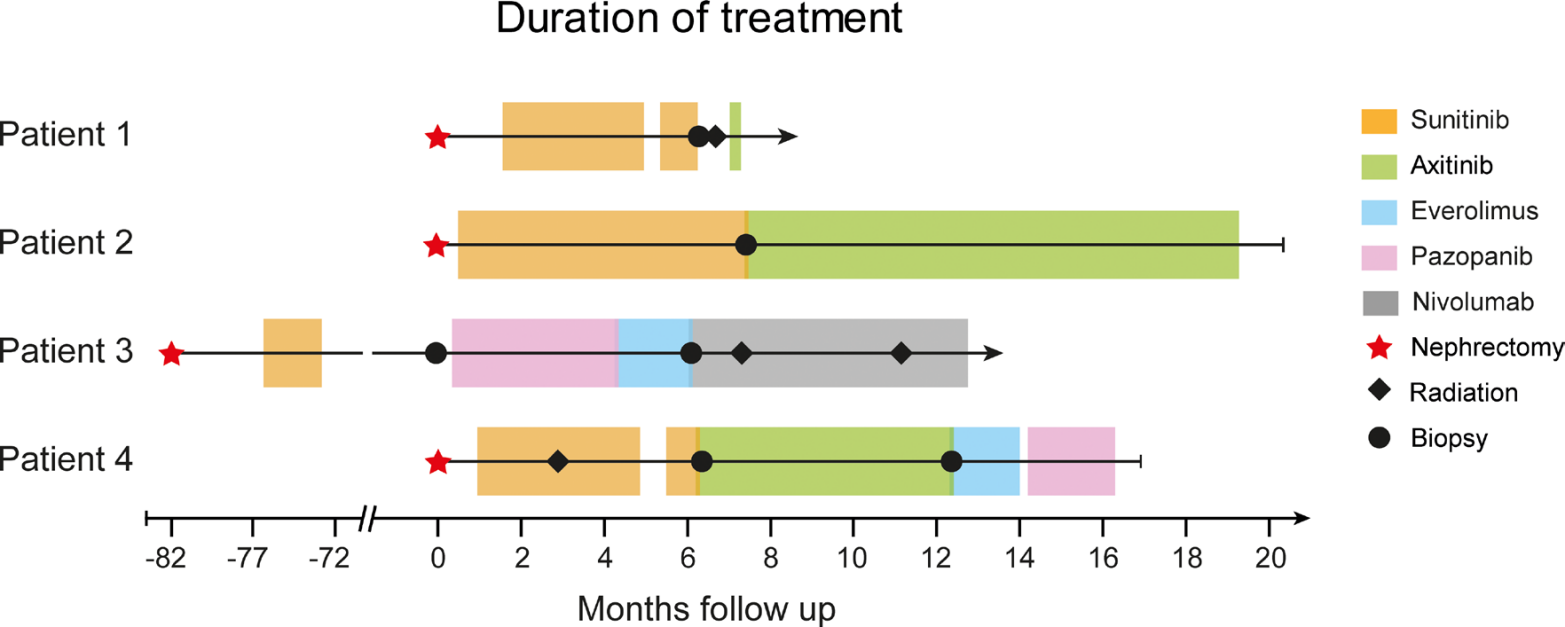
Swimmer plot with therapy sequences and durations of different treatment lines for each patient The different therapies are indicated by colors and include nephrectomy, radiation therapy, and biopsies from progressive sites.

Four of the 17 patients enrolled in the MORE study (Table 1) showed progression under TKI treatment. At these time points, metastasis biopsies were collected, and DNA from tumor tissues at baseline and progression was subjected to WES. Patient 1 (female, 81.3 years) progressed under sunitinib therapy after 5.4 months, and a biopsy (vaginal wall metastasis) was taken (Figure 1). Patient 2 (female, 70.5 years) progressed after 6.2 months under sunitinib therapy. After needle biopsy from the right chest wall, the therapy was continued with axitinib until she suffered a second progression after 11.8 months (Figure 1). Patient 3 (male, 46.3 years) showed no residual malignant disease after cytoreductive nephrectomy and adjuvant sunitinib treatment. At relapse after 6.1 years (73 months), a baseline biopsy of the left chest wall metastasis was taken, and treatment was continued with pazopanib. He progressed at 3.9 months and the therapy was switched to everolimus, which was discontinued after 1.8 months due to clinical progression (soft tissue metastasis). After re-biopsy of the chest wall metastasis, nivolumab treatment was initiated (Figure 1). Patient 4 (male, 58.9 years), progressed after 5.3 months sunitinib treatment, and a biopsy was collected from the os ilium. In addition, a cutaneous nodule in the left chest wall could be resected when he progressed after 6 months on axitinib (Figure 1). The therapy was continued with everolimus for 1.5 months, followed by pazopanib for 2 months. The patient died of the disease soon thereafter.

**Table 1:** Clinical characteristics of the four patients who experienced tumor progression

	Patient 1	Patient 2	Patient 3	Patient 4
**Age (y)**	81.3	70.5	46.3	46.3
**Gender**	F	F	M	M
**TNM**	T4N2M1	T1aN0M1	T3bN2M1	T3aN2M1
**Fuhrman Grade**	G3	G2	G2	G3
**Site of metastasis**	paraaortal lymph node, lung, bilataral adrenal, cava thrombus	lung, bone, liver	retroperineal lymph node, bone, chest wall, pleura	lung, bone, adrenal

### Molecular analysis at baseline and tumor progression

**Figure 2:**
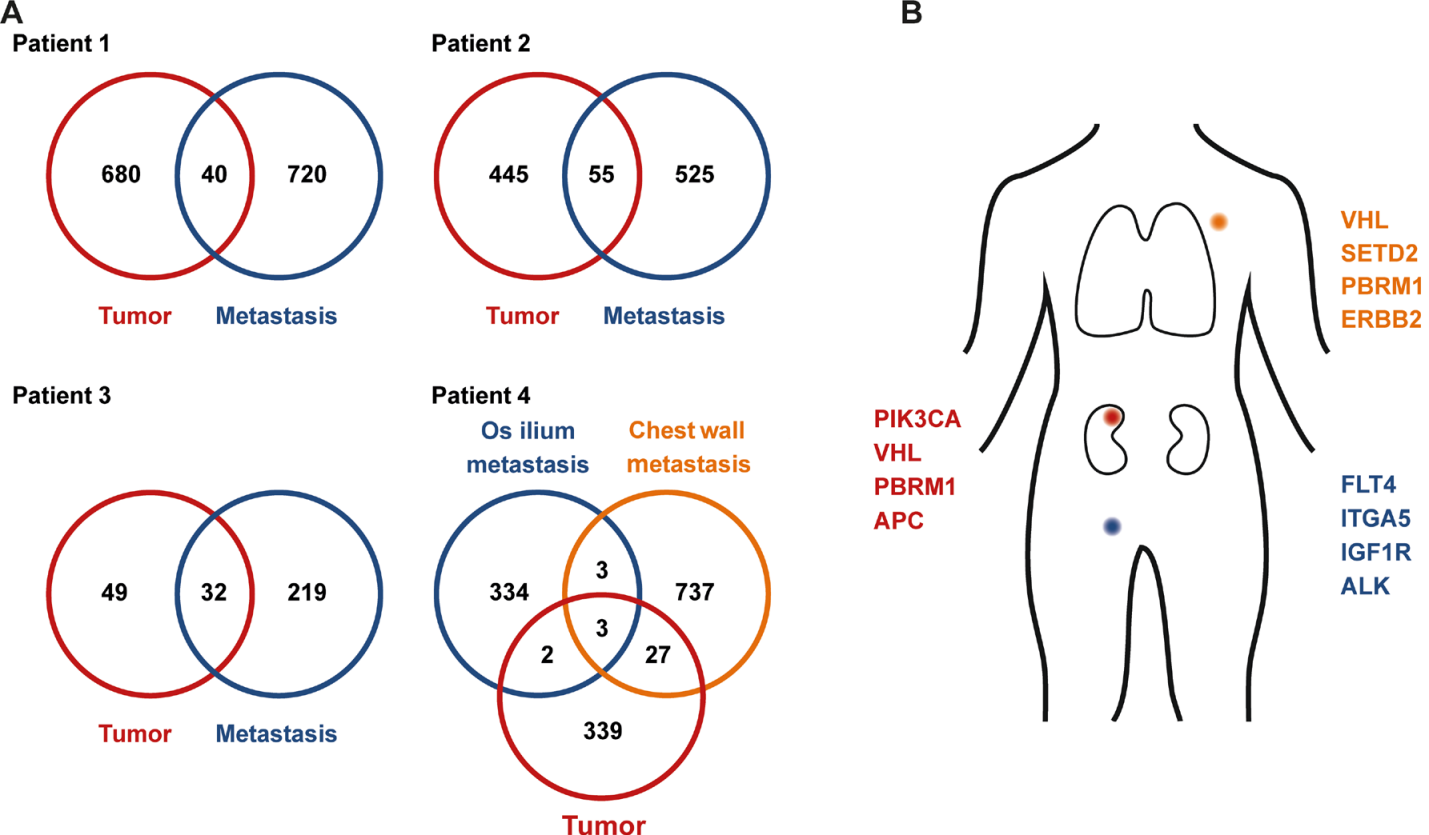
Clonal mutations in primary and metastatic sites Numbers of exclusive and shared mutations identified in primary tumor tissue and metastasis biopsies (**A**). Coding mutations in the primary tumor and metastasis biopsies from patient 4 (**B**).

**Figure 3:**
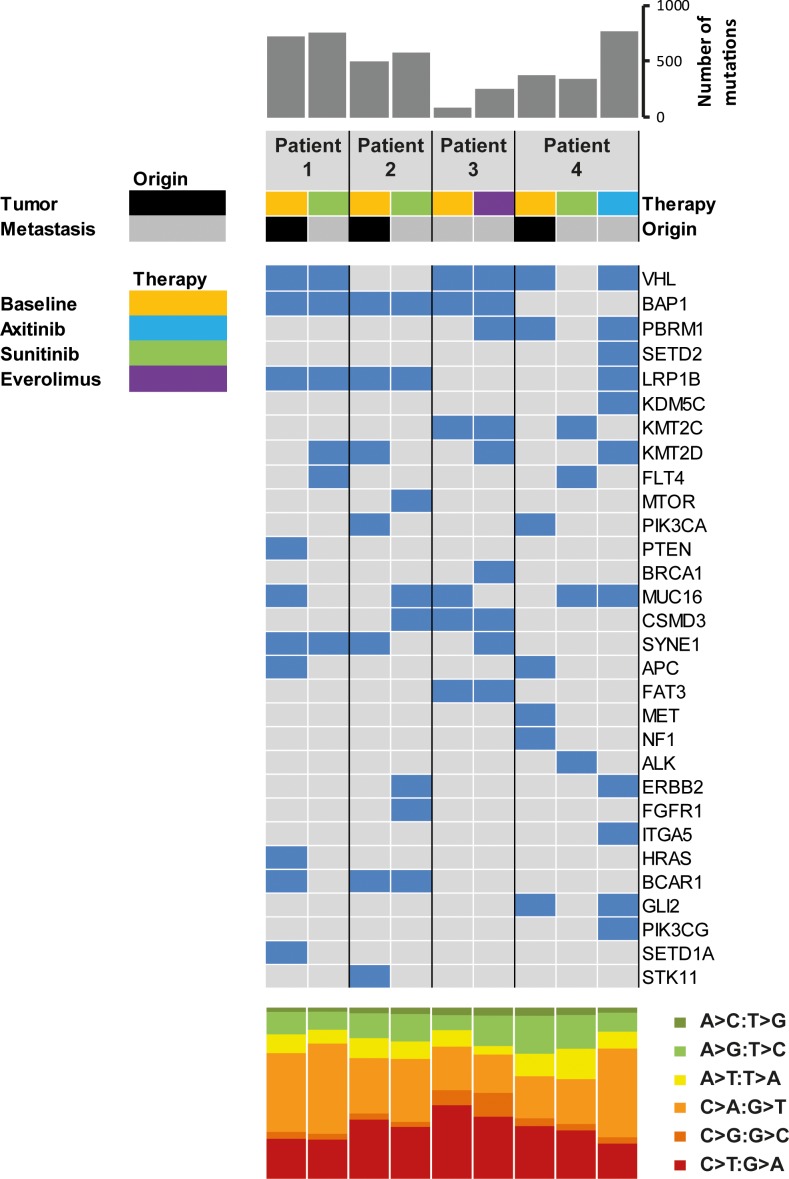
Coding somatic mutations identified in primary tumors and metastatic sites at baseline and upon progression The upper histogram shows the number of nonsynonymous mutations per sample. The heatmap indicates the presence (blue) and absence (grey) of mutations in selected cancer-associated genes in primary and progressive samples of the four patients. The lower histogram shows the proportions of base substitutions for each sample.

To evaluate clonal diversity and molecular alterations upon progression on TKI treatment of the four patients (Nr. 1-4), we performed WES of the primary tumor and biopsy samples from metastatic sites upon disease progression (Figure 1). Matched buffy coat samples were sequenced to exclude germline alterations from sequencing data. Sequencing of tumor and and matched buffy coat resulted in a mean target coverage of 124× and revealed an average of 486 non synonymous somatic mutations (Table 2, Supplementary Table 1) with a mean coverage of 104× at mutation sites. Mutations in RCC-associated genes, including *VHL*, *BAP1*, *PBRM1*, *LRP1B*, and *KMT2C* were further validated by Sanger sequencing. Consistent with previous reports [16, 17], the genetic compositions among the four patients were diverse (Figure 2), with alterations in genes known to be recurrently mutated in ccRCC, i.e. *VHL*, *SETD2*, *PBRM1*, and *BAP1* [14, 15] and others which are known oncogenes or might be potential therapy targets. These selected genes are shown in Figure 3. All somatic nonsynonymous mutations and their resulting effect on transcripts and proteins are given in Supplementary Table 1.

**Table 2:** Sample characteristics and quality metrics of the sequencing data

Case	Type	Timepoint	Site	Target coverage [x]	Mapped Reads [%]	Insert Size [bp]	Functional somatic SNVs
**Patient 1**	Tumor	Baseline	right kidney	106.03	86.11	166	720
	Metastasis	1. Progress	left vaginal wall	121.8	85.63	169	760
	Buffy Coat	Baseline	blood	109.93	88.83	171	-
**Patient 2**	Tumor	Baseline	left kidney	111.81	85.38	172	500
	Metastasis	1. Progress	right chest wall	110.09	88.96	174	580
	Buffy Coat	Baseline	blood	117.94	88.56	173	-
**Patient 3**	Metastasis 1	Baseline	left chest wall	156.9	97.73	184	81
	Metastasis 2	1. Progress	left chest wall	151.96	98.34	164	251
	Buffy Coat	Baseline	blood	158.27	97.95	164	-
**Patient 4**	Tumor	Baseline	left kidney	109.85	88.33	172	371
	Metastasis 1	1. Progress	os ilium	117.01	90.06	176	342
	Metastasis 2	2. Progress	left chest wall	126.32	87.42	167	770
	BuffyCoat	Baseline	blood	120.94	90.53	174	-
			**Mean**	**124.53**	**90.29**	**171.23**	**486**

We found remarkable heterogeneity between primary and metastatic lesions with only a small subset of alterations present in both sites (Figure 2): WES of the primary tumor from patient 1 identified private mutations in the tumor suppressor genes *PTEN* and *APC* as well as in *HRAS, BCAR1* (COSM1479104), and *SETD1A* (COSM126103). Forty mutations, including in *VHL* (COSM14311), *BAP1,* and *STK25* were shared between primary and metastatic site (Figure 3, Patient 1). The metastasis upon progression under sunitinib carried mutations in *FLT4*, *KMT2D*, and *BMP5*, which were not detected in the primary tumor. We identified 55 mutations shared between primary tumor and metastasis of patient 2 (Figure 2A), including *BAP1, LRP1B,* and *BCAR1*. She had a private mutation in *PIK3CA* (COSM1041490) in the primary tumor. In line with previous findings suggesting that *mTOR* mutations occur predominantly at advanced stages of tumor evolution in subclonal metastatic populations [16, 17], the metastasis revealed a private *mTOR* mutation. Additional mutations were found in *FGFR1* and *ERBB2* (Figure 2). After cytoreductive nephrectomy and adrenalectomy of the left kidney, patient 3 was treated with sunitib and showed no evidence of residual disease or indications of progression during follow-up. When the tumor relapsed after more than six years with synchronous bone, pleural and chest wall metastases, a biopsy from the left chest wall metastasis was used as a baseline sample. After progression on pazopanib and everolimus, this metastasis was re-biopsied, and the therapy was continued with nivolumab. WES analysis showed an increase of mutational load between baseline (81 mutations) and progression (251 mutations), and 32 (39.5%) of the 81 baseline mutations were also found in the re-biopsy (Figure 3), including changes in *VHL*, *BAP1*, *KMT2C*, *CSMD3*, and *FAT3*. The re-biopsy revealed additional private mutations in the epigenetic regulators *KMT2D* (COSM1299437) and *KMT2E* (COSM1083684) as well as in *PBRM1*, one of the most frequently mutated genes in ccRCC, which codes for a subunit of the BAF chromatin remodeling complex. Furthermore, we detected a mutation in *BRCA1*, which was not been present at baseline. In patient 4, WES of the primary tumor and two different locations of metastases was performed (Figure 2A, 2B). Only three (*RYR3*, *ABI3BP*, and *TMEM255A*) of the 1445 identified nonsynonymous somatic mutations were found to be present at every site. Notably, the mutational spectrum of the primary tumor revealed a larger overlap with the chest wall metastasis than with the initial pelvic bone metastasis (30 vs. 5 mutations, respectively), including changes in *VHL* and *PBRM1,* possibly indicating an early separation of the cells giving rise to the latter metastasis. The analysis of the chest wall metastasis identified further mutations in cancer-associated genes, including *SETD2* and *ERBB2*. In addition, with 700 somatic variants, the mutational load of the chest wall metastasis was considerably higher than in the primary tumor and the metastasis located in the os ilium (371 and 342 mutations, respectively). This suggests an early separation of cells giving rise to the chest wall lesion.

## DISCUSSION

The current pharmacological treatment strategies in ccRCC are based on targeted drugs and, more recently, immune checkpoint inhibitors. Although numerous TKIs are available, tumor heterogeneity and secondary resistance are major challenges for cancer therapy. Especially a heterogeneous phenotypic response to TKI therapy and progression has recently been found [19]. Hence, a deeper understanding of the complexity of molecular and mutational signatures of ccRCC is needed. This can be achieved with the ultra-high DNA sequencing. However, apart from large catalogues of somatic mutations in cancer including ccRCC [14, 15] provided by international sequencing projects like TCGA and ICGC, multiregional sequencing also revealed remarkable intra-tumor heterogeneity [16–18, 20] Here, we provide evidence for considerable temporal molecular heterogeneity between therapy-naïve primary tumors and metastases developing under TKI. The mutational profiles provide insights into clonal evolution occuring during tumor progression under therapy: Molecular profiling revealed recurrent genomic alterations in genes frequently altered in ccRCC [14], including *VHL*, *SETD2*, and *BAP1.* Deletions of the *VHL* tumor suppressor gene are occuring early during tumorigenesis of ccRCC [16]. We detected additional *VHL* mutations in baseline samples and metastatic sites in three out of four patients. Well-known alterations were found to be common to primary and metastatic sites. These included mutations in the ccRCC tumor-driving gene *BAP1* in patients 1, 2, and 3. The clonality of the *BAP1* mutation, which has been associated with poor prognosis and a high metastasizing potential [21] supports *BAP1* as a molecular marker for ccRCC sub-classification [21].

In agreement with previous reports [16, 17], our WES results revealed that *mTOR* mutations occur predominately in subclonal branches in advanced disease stages (patient 2). We also identified two different *ITGA5* mutations in independent metastases, but not in the primary tumor, of patient 4, suggesting parallel evolution of the two metastatic clones. In contrast to patients 1–3, most of the mutations in patient 4 were found to be private for each sample. This might be due to several reasons: Either the initial molecular features present in the primary tumor were lost in the metastases, or multiple novel mutations accumulated in the metastases, or the sequenced tissue region in primary tumor did not contain the clones that gave rise to the metastases. Although we cannot distinguish between these possibilities, all of them are consistent with a high molecular heterogeneity present early during tumor development, followed by clonal selection and/or independent evolution after dissemination of tumor cells. Supported by previous findings in breast cancer [22], the larger overlap of the primary tumor with the chest wall metastasis than with the initial pelvic bone metastasis possibly indicates an early separation of the cells giving rise to the latter metastasis.

The identification of clinically relevant mutations upon tumor progression under TKI treatment suggests that it might be possible to derive alternative targeted therapies based on molecular changes in the metastases. The analysis of the molecular patterns upon TKI progression revealed different potentially targetable mutations in all cases tested. While we detected subclonal mutations in *FLT4*, *ITGA5, SETD2,* and *BRCA1* in the metastases, one patient developed mutations in *mTOR* and several receptor tyrosine kinase genes, including *ERBB2*, *ERBB4*, and *FGFR1*. Moreover, the chest wall re-biopsy of patient 3 exhibited mutations in *PBRM1* and *BAP1*, which are mostly mutually exclusive. Their co-occurrence has been associated with aggressive tumors and poor prognosis [23, 24]. The acquired *BRCA1* mutation, which was not detected in the baseline of patient 3, represents another impairment of DNA repair, which might indicate that PARP inhibitors could be beneficial in this case. Activity of PARP in *BRCA*-mutated ovarian and prostate cancer is well established and olaparib is clinically approved [25, 26]. Activity of PARP inhibitors in *BRCA*-mutated ccRCC has not yet been reported. However, the molecular principle remains similar through different entities, and an individual treatment approach after failure of approved substances may be warranted. Alternatively, *BRCA1* mutations giving rise to impaired DNA repair may result in increased expression of neoantigens, a potential marker of sensitivity to immune checkpoint inhibitors. Furthermore, VEGFR3/FLT4 inhibitors that are currently in early clinical development may be active in patients carrying *VEGFR3*/*FLT4* mutations, respectively [27]. Mutations in *mTOR* may be particularly sensitive to mTOR inhibitory drugs. Finally, several drugs targeting the FGFR are currently under clinical development.

To the best of our knowledge, this study is the first to examine molecular alterations associated with ccRCC therapy resistance in tissue DNA in a longitudinal fashion. The molecular data provide evidence for various routes leading to drug resistance of ccRCC subclones with acquired mutations in known therapy target genes on metastatic progression.

The major limitation of our study is its small sample size due to the low progression rate to date, and our findings have to be corroborated in larger studies. To this end, MORE is open and actively recruiting, and we will provide an extension of the current data set in due time. However, our results agree with comparable precision medicine approaches in other tumor entities in that the progression of patients under therapy is very specific and restricted to the individual case. In other words, highly recurrent targetable mutations cannot be expected from the analysis of many more cases. In contrast, the serial molecular analysis of tumors from individual ccRCC patients progressing under therapy might indicate ways leading to therapy resistance and support tailored treatment decisions.

## MATERIALS AND METHODS

### The MORE protocol

The MORE (Molecular Renal Cancer Evolution) study is a prospective clinical study designed to investigate molecular alterations in metastatic ccRCC progressing under first-line TKI. The primary objective of MORE is to characterize molecular alterations in metastatic lesions compared to baseline tissue in order to understand the individual factors leading to therapy resistance and tumor progression. Secondly, we hope to identify potential molecular targets for personalized therapies for the progressive patients in order to improve individual patient care. The study was approved by the ethics committee at the Heidelberg University Medical Faculty (S-539/2013) and listed on clinicaltrials.gov (NCT02208128) and the national study register (DRKS0006193). Patients with metastatic disease and no prior systemic therapy were eligible for the study. At baseline, tissue samples from debulking primary tumor surgery or biopsy samples were taken. After standard of care first and second line therapies, biopsies of progressing metastases were sampled.

### Patients and samples

Seventeen patients were classified as low risk according to the MSKCC [8] prognostic score and had pure clear cell histology at baseline and progression. These included 11 male and six female patients with a median age of 65 years (range: 46 to 82 years) at the date of initial diagnosis. Four patients had initial tumor stage T1, one patient T2, nine patients T3, and two patients T4 disease. Sixteen patients had already developed metastatic disease at diagnosis. Of the 17 recruited patients, four progressed under TKI treatment and were subjected to the molecular analyses (Table 1). Selection criteria for these four patients were the disease progression and the availability of high quality DNA from progressive metastases. Six primary tumor and three normal adjacent tissue specimens from each patient were taken upon resection and immediately frozen in liquid nitrogen. Biopsies were taken during tumor progression. The histology of all tissue samples were confirmed by a board-certified pathologist. Unstained and unembedded samples with a median tumor cell fraction of 70% (range: 40–90%) were subjected to DNA isolation, followed by WES.

### Isolation, quantification, and quality control of genomic DNA

Fresh frozen surgical tumor tissue and biopsy samples (10–30 mg) were mechanically disrupted and homogenized using the TissueRuptor (Qiagen, Hilden, Germany). Genomic DNA was extracted using the QIAamp DNA Mini Kit (Qiagen) according to the manufacturer’s protocol. As germline control, genomic DNA from matched blood cells was isolated using the QIAamp DNA Blood Mini Kit (Qiagen). DNA concentrations were determined using the Qubit (Thermo Fisher Scientific, Waltham, MA, USA), and DNA integrity was assessed using the TapeStation (Agilent Technologies, Santa Clara, CA, USA).

### Library construction and exome sequencing

Sequencing libraries were prepared from 200 ng genomic DNA. Prior to library preparation, all DNA samples were sheared to an average fragment length of 150 bp using the Covaris S220 ultrasonicator. Exome-enriched sequencing libraries were prepared using the Agilent SureSelectXT Human All Exon V5+UTR kit (low input protocol). Library sizes and qualities were evaluated before and after capture by Bioanalyzer 2100 analysis using the High Sensitivity DNA Kit (Agilent) and quantified using the Qubit dsDNA HS Assay kit (Thermo Fisher Scientific). All libraries were subjected to 100 bp paired-end sequencing on the Illumina HiSeq 2000 v3 at the DKFZ Genomics and Proteomics Core Facility. Selected variants were further validated by bidirectional Sanger sequencing on an ABI 3130 Genetic Analyzer (Thermo Fisher Scientific, Waltham, USA) using the BigDye Terminator v1.1 Cycle Sequencing Kit (Thermo Fisher Scientific, Waltham, USA) as described previously [28].

### NGS data analysis and variant calling

Sequence data analysis and mutation calling was performed using the One Touch Pipeline (OTP), a fully automated computational platform for sequence analysis. Briefly, raw FASTQ data files and metadata were loaded into OTP. After quality score estimation using FastQC, the files were aligned to the human genome (hg19) using BWA [29]. All aligned files were subsequently merged with Picard. Files belonging to one same sample were combined into one file, which was quality controlled using a variety of in-house tools and standard SAMtools [30]. PCR duplicates were removed, and only unique reads were used for variant calling and target coverage estimation. After processing of bam files, an in-house SNV calling pipeline, consisting of three different steps (calling, annotation, and filtering) was used based on each tumor-control comparison. For variant calling, samtools, mpileup, and bcftools were used. Called variants were annotated using the dbSNP and COSMIC databases and filtered for somatic variants in protein coding regions with a mutant allele frequency of > 2.5%. If a mutation was found in only one of the matched primary/metastatic samples from the same patient, unfiltered somatic and non synonymous exonic variants were called independent of their allele frequencies.

### Data availability

FASTQ files are available at the European Genome-Phenome Archive (https://ega-archive.org/) under the accession number EGAS00001001861.

## SUPPLEMENTARY MATERIALS TABLE





## Abbreviations

ccRCC: clear cell renal cell carcinoma
MORE: Molecular Renal Cancer Evolution
mRCC: metastatic renal cell carcinoma
OS: overall survival
OTP: One Touch Pipeline
SD: stable disease
TKI: tyrosine kinase inhibitor
VEGF: vascular endothelial growth factor
WES: whole-exome sequencing
