# Supplementary material for: Patient-specific molecular alterations are associated with metastatic clear cell renal cell cancer progressing under tyrosine kinase inhibitor therapy
Source: Oncotarget. 2017 May 23;8(43):74049–57. doi: 10.18632/oncotarget.18200 (PMC5650322; doi:10.18632/oncotarget.18200)
Supplement: Supplementary file 1 [file oncotarget-08-74049-s001.pdf]

Patient-specific molecular alterations are associated with metastatic clear cell renal cell cancer progressing under tyrosine kinase inhibitor therapy

SUPPLEMENTARY MATERIALS

Supplementary Table 1: Somatic nonsynonymous mutations identified in patient sample.
See Supplementary Table 1
